# The National Medical Guild: A Cash Alternative to Medical Benefit

**Published:** 1913-09-27

**Authors:** M. Milton Townsend

**Affiliations:** Member Council Nat. Med. Guild.


### THE NATIONAL MEDICAL GUILD.

President: CHAS. BUTTAR, M.A., M.D., B.C.

Gen. Sec.: P. C. RAIMENT, M.R.C.S., L.R.C.P.

Offices: 34 Villiers Street, Strand, London, W.C.

The other day we received from Dr. Milton Townsend the paper which we print below, and we thought it of sufficient merit to deserve publication here. Whilst offering no comments on it, we shall be glad to hear from the members any criticisms or suggestions that may occur to them on reading it.

P. C. R.

### A CASH ALTERNATIVE TO MEDICAL BENEFIT.

### A Scheme for Allowing Persons Insured under the National Insurance Act to make their own Arrangements for Medical Benefit, receiving in lieu thereof a Monetary Equivalent.

By M. MILTON TOWNSEND, L.R.C.S., L.R.C.P. (Edin.), Member Council Nat. Med. Guild.

In considering this scheme it must be borne in mind that notwithstanding any questions of policy or tactics which have up to the present caused the administrators of the Act to refuse to insured persons, save under very special circumstances, permission to make their own arrangements for medical benefit, it was clearly in the minds of the framers of the Act that there would be many persons who would either not be permitted or would not desire to avail themselves of its medical benefits. This is clearly shown in Section 15, paragraph 3, which runs as follows:—

"The regulations made by the Insurance Commissioners shall authorise the Insurance Committee by which medical benefit is administered (i) to require any persons whose income exceeds a limit to be fixed by the Committee, and (ii) to allow any other persons in lieu of receiving medical benefit under such arrangements as aforesaid to make their own arrangements for receiving medical attendance and treatment (including medicines and appliances), and in such case the Committee shall, subject to the regulations, contribute from the funds out of which medical benefit is payable towards the cost of medical attendance and treatment (including medicines and appliances) for such persons sums not exceeding in the aggregate the amounts which the Committee would otherwise have expended in providing medical benefit for them."

Mr. A. S. Comyns Carr, in his book on National Insurance, which work has received the approbation of the Chancellor in the following terms, "I heartily commend the book to all who wish to bear their share in working out the scheme which Parliament has initiated," makes the following comments on this section: "(i) The Commissioners are under compulsion to authorise in their regulations the Insurance Committees, but the Committees are not under compulsion to fix any income limit, though by Section 62 they are bound to consult the local Medical Committees when the question of fixing an income limit arises." This is equally applicable to the District Committees which have been recently formed in London under Section 59 (4), and under Section 62 it is equally possible for local Medical Committees to be formed corresponding to the area of the District Committees, and to have the rights granted to them in the Act and regulations (Section 62, Regulation for Medical Benefit, Part V., Section 53).

It was, therefore, clearly the intention of the Act that every Insurance area should be able to fix its own income limit, and that insured persons whose income exceeded that limit should be required to make their own arrangements for medical benefit, including the supply of medicines and appliances.

(ii) "Allow any other persons"—that is, irrespective of income limit. Primarily intended to meet the case of insured persons who desire to be medically attended by practitioners who are not included in the panel.

The inference from this paragraph ((iii) Section 15) is obvious, and any departure from it can only be construed as a policy by those responsible for the administration of the Act of attempting to coerce both insured persons and doctors into the panel system, which in a large number of cases is distasteful to both, and is not necessary for the efficient working of the medical provisions of the Act. The only logical deduction to be drawn from Section 15 (iii), which is known as the Addison Amendment, and which was proposed by Dr. Addison in the House of Commons after consultation with the B.M.A. (Comyns Carr), is that it was within the knowledge of the framers of the Act that there were a large number both of insured persons and medical men to whom any form of medical service under the Act would be unsuitable, and that provision must be made in the Act for dealing with these cases in some manner satisfactory to both parties.

Whilst freely admitting that for the majority of the insured some form of contract medical attendance is the only feasible way of carrying out the medical provisions of the Act, I would point out the fact that prior to the passage of this Act under 6,000,000 people were receiving medical attendance at contract rates, and as practically every one of the present 15,000,000 insured persons had the opportunity, had they wished, of receiving through any one of the numerous friendly societies, sick benefit clubs *et hoc genus omne*, medical attendance at contract rates, we may fairly assume that they did not avail themselves of this opportunity because they had no desire to do so, and that as there are, so far as the patient is concerned, no material advantages to him in the present panel system over the old club system, one may judge that their objection to contract medical attendance still holds good. In the same way a large number of medical men have in the past, for personal or professional reasons, steadfastly set their faces against accepting any form of contract work; and, again, I do not see that the panel or any other system at present in vogue of attending patients under the Insurance Act offers them any inducement to change their minds.

In view of the facts, first, that there is a section of the Act expressly inserted for the use of those insured persons who either may not or do not wish to avail themselves of medical benefits, and, secondly, there are several thousand insured persons who, although medical benefits have been accessible for six months, have not attempted to avail themselves of them, it is the duty of those entrusted with the administration of the Act to put into force the section and regulations which enable the insured to make their own arrangements for medical treatment.

Paragraph 22. Conference with the Chancellor of the Exchequer, B.M.J. Supplement, December 7, 1912, distinctly sanctions the principle of contracting-out . . . "It will still remain possible for any insured person who so desires to make his own arrangements with an individual practitioner (whether on the panel or not) on terms under which he will be liable to pay to the doctor such amount, if any, as the doctor charges for attendance and treatment (apart from drugs) during the year beyond the amount available from the medical benefit fund."

This statement does not entirely coincide with Section 15 (iii), in which own arrangements are to include medicines and appliances, but it is of the greatest importance as showing that there was no intention, even on December 7 last, to bar contracting-out with doctors not on the panel.

I submit that the following points are clearly established:—

(*a*) That free contracting-out is an integral portion of the Act provided for by statute and regulations.

(*b*) That, subject to the approval of the Insurance Committee, contracting-out is open to any insured person, and inferentially permission should not be withheld for the purpose of coercing practitioners on the panel.

(*c*) Persons who contract out are at liberty to obtain their medical attendance from practitioners either on or off the panel.

As about 1,400 medical men in London are on the panel, there are now no logical reasons for refusing to give the widest interpretation to the section in question, and proceeding to formulate a scheme by which contracting-out patients can consult any doctor they may desire. It remains, then, to devise a scheme which will carry out the provisions of Regulations, Part II., Regulation 14, Paragraph 7: "The Committee may allow any insured person resident in the County, whether individually or collectively, in lieu of receiving medical benefit under the arrangements made by the Committee, to make their own arrangements for receiving treatment (including medicines and appliances)."

I am aware that there is already in existence a pooling scheme for the two or three hundred persons in London who have been allowed to contract out, but this scheme is open to many objections.

(i) It fixes a maximum scale of fees, and thereby limits the number of medical men willing to attend patients in the pool to those whose ordinary fees are less than, or no greater than, the scale fees allowed by the pool.

(ii) By proportionate payment of bills out of a pool there is the inevitable desire of the subscribers to get their money's worth, with the consequence that the doctor who sets his face sternly against needless visits or attendances is at a great disadvantage with those who are not of so stern a moral calibre. It is, in fact, a bad combination of an insurance and a gamble, and from an ethical point of view compares unfavourably with the simple capitation system. It must not be forgotten that the insured person who wishes to contract out of medical benefit has presumably in the past been able and willing to pay his medical bills, and, provided that he suffers no financial loss under the Act, he is still able and willing to go on paying. Therefore, return to him the monetary equivalent of the amount due to him for medical benefit and allow him to proceed in the future as he has in the past, only regulating the method of returning him his monetary equivalent so that it is only available for the purpose of receiving medical benefit.

The method I would suggest is that each person contracting-out should receive quarterly, providing his contributions entitled him to it, a coupon or ticket bearing the face-value of the amount payable for that quarter for medical benefit in respect of any person who is receiving treatment on the panel system *plus* amount available for medicines and appliances during that quarter. For example, the amount payable to the practitioners on the London panel is 1s. 9d. per quarter, and 6d. per quarter is set aside for drugs, total 2s. 3d. The ticket in this case would bear the face-value of that amount. It would be a non-transferable ticket made out in the name of the insured person, with a space for his signature, and would be available at any time during the year of issue. On presenting himself for treatment the doctor would take the ticket in whole or part payment, according to his usual fees, and he could cash it at suitable times at the offices of the London Insurance Committee. Any further treatment during the quarter would be payable by the insured person. If at the end of a year the insured had had no illness his credit tickets would be carried forward to the next year, and so on. As these tickets would be cashed on demand the insured would be debited with the quarterly amount in exactly the same way as would be done were he receiving attendance on contract. On surrendering his ticket in exchange for medical attendance his signature would be a receipt for the attendance, and by this means trafficking in tickets could be checked.

**(Continued on page 3 of "Institutional Worker."**

